# Mapping TriNetX-Based Real-World Evidence Publications by Clinical Domain and Study Purpose, 2018–2025: A Bibliometric Analysis

**DOI:** 10.3390/healthcare14142143

**Published:** 2026-07-16

**Authors:** Min-Chih Hsieh, Ming-Chi Lu, Malcolm Koo

**Affiliations:** 1 Division of Obstetrics and Gynecology, Dalin Tzu Chi Hospital, Buddhist Tzu Chi Medical Foundation, Dalin, Chiayi 622401, Taiwan; 2 Division of Allergy, Immunology and Rheumatology, Dalin Tzu Chi Hospital, Buddhist Tzu Chi Medical Foundation, Dalin, Chiayi 622401, Taiwan; 3 School of Medicine, Tzu Chi University, Hualien City 970374, Taiwan; 4 Department of Medical Research, Dalin Tzu Chi Hospital, Buddhist Tzu Chi Medical Foundation, Dalin, Chiayi 622401, Taiwan; 5 Dalla Lana School of Public Health, University of Toronto, Toronto, ON M5T 3M7, Canada

**Keywords:** TriNetX, real-world evidence, electronic health records, federated data networks, bibliometric analysis, clinical domain mapping, author keywords, research trends

## Abstract

**Background/Objectives**: Federated electronic health record networks are increasingly used for real-world evidence generation, but publication-use patterns for TriNetX remain incompletely characterized. This study mapped Web of Science-indexed articles that explicitly mentioned TriNetX and examined their distribution across clinical domains and study purposes. **Methods**: We searched the Web of Science Core Collection for articles published during 2018–2025 with “TriNetX” in the title, abstract, or author keywords. Bibliometric indicators, journal sources, citation impact, author-keyword co-occurrence, and trend topics were analyzed using R, bibliometrix, Biblioshiny, and VOSviewer. A reproducible rule-based dictionary classified clinical domains and study purposes, and a supplementary Scopus analysis assessed pattern-level reproducibility. **Results**: The corpus included 1573 articles. Annual output increased from 1 article in 2018 to 871 in 2025. Corresponding-author output was concentrated in the United States and Taiwan. Highly cited studies were dominated by COVID-19 research. Keyword analyses suggested a shift from pandemic-related topics toward mortality, cardiometabolic disease, and medication-related outcomes. Domain–purpose mapping showed that risk/prognosis studies were most common, whereas effectiveness, health services, and method/validation studies were less frequent. Scopus validation reproduced the main annual, source-level, and domain–purpose patterns. **Conclusions**: TriNetX-based publications expanded rapidly and diversified across clinical fields. Domain–purpose mapping shows where research has concentrated and where it remains sparse; lower frequency, however, should not be read as a definitive clinical evidence gap, since some areas may be less suited to the platform. Where the network is well suited, future work could give greater attention to comparative effectiveness, long-term safety, care pathways, and phenotype validation.

## 1. Introduction

Secondary data analysis has become central to contemporary clinical evidence generation. Data sources such as government surveys, insurance claims, and electronic health records (EHRs) allow cost-efficient analyses of large populations, including rare outcomes and long-term trajectories [1]. However, conventional databases, including Taiwan’s National Health Insurance Research Database (NHIRD) [2] and the United States National Health and Nutrition Examination Survey (NHANES) [3], can be limited by incomplete clinical detail, coding inaccuracies, and rigid data structures.

EHR-based platforms help address these gaps by providing granular clinical information generated at the point of care. TriNetX (https://trinetx.com/), launched in 2013, is a prominent federated health data network that enables privacy-preserving analyses of de-identified patient data across participating healthcare organizations. By harmonizing heterogeneous EHR data from a large multinational patient population, TriNetX supports large-scale observational research, trial optimization, cohort discovery, and analyses incorporating diagnoses, procedures, medications, laboratory results, and vital signs, with potential linkage to external sources such as claims and mortality records [4,5,6]. Its scale, clinical depth, and rapid analytic capability have made it an important infrastructure for real-world evidence generation, although findings require interpretation in light of hospital-based selection bias, cross-institutional heterogeneity in data capture, and constraints imposed by the platform’s fixed analytic interface [7].

The platform has been rapidly adopted, with publications spanning infectious diseases, chronic conditions, and health outcomes research [8]. During the coronavirus disease 2019 (COVID-19) pandemic, TriNetX provided near real-time evidence for risk stratification and treatment evaluation [9]. As the pandemic receded, its application has broadened to chronic disease management [10] and long-term outcomes [11]. This shift suggests that TriNetX has moved from an emergency-response data source toward a more general infrastructure for real-world evidence generation.

TriNetX has been examined in comprehensive reviews and region-specific bibliometric analyses. One review described its analytical methodologies and broad clinical applications, emphasizing its potential to complement randomized controlled trials [6]. Another critically assessed its methodological framework, strengths, and limitations for real-world evidence generation [12]. A bibliometric study restricted to 92 publications with Taiwan-affiliated authors described local research output and institutional contributions, but its regional scope limited evaluation of global publication patterns [13]. Collectively, these studies show the growing importance of TriNetX and the need for a comprehensive analysis of its worldwide research landscape.

Despite this rapid expansion, the publication landscape of Web of Science (WoS)-indexed articles explicitly mentioning TriNetX remains incompletely characterized. Recent studies have begun to describe TriNetX-related publications, including country-specific and PubMed-based analyses, but a comprehensive mapping of Web of Science-indexed original articles across countries, institutions, publication sources, citation patterns, thematic structures, and clinical domain–study purpose combinations remains lacking. Bibliometric approaches can provide this overview by helping researchers situate new work within the existing evidence base, identify underexplored areas, and reduce duplication of effort [14]. For federated electronic health record networks, such mapping is particularly useful because it can show how real-world evidence infrastructure is being used across clinical domains, where research activity is concentrated, and where healthcare data platforms remain underused.

The present study therefore provides a bibliometric overview of WoS-indexed original articles published from 2018 to 2025 that explicitly mentioned TriNetX in the title, abstract, or author keywords, and applies a reproducible clinical domain–study purpose mapping approach to examine how the platform has been used across clinical fields within this search-defined corpus. This article-level mapping extends beyond the outputs of generic bibliometric software and provides a more practice-oriented basis for identifying research gaps in TriNetX-based real-world evidence generation.

## 2. Materials and Methods

### 2.1. Data Source, Search Strategy, and Record Selection

The WoS Core Collection, Science Citation Index Expanded (Clarivate Analytics, Philadelphia, PA, USA), was searched on 18 May 2026. This database was selected because it is widely used in bibliometric research and provides broad coverage of influential scientific journals as well as complete cited reference information required for citation-based analyses [15]. The primary search used a title–abstract–keyword search strategy to identify records in which “TriNetX” appeared in the title, abstract, or author keywords: TI = (TriNetX) OR AB = (TriNetX) OR AK = (TriNetX). This strategy defined the analytic corpus as WoS-indexed articles that explicitly named TriNetX in the selected searchable fields; it was not intended to identify articles that may have used TriNetX without naming the platform in the title, abstract, or author keywords. This search retrieved 2915 records. As a sensitivity check, a broader Topic Search (TS), TS = (TriNetX), was also performed and retrieved the same number of records, indicating that the inclusion of Keywords Plus did not change the retrieval count.

**Figure 1:**
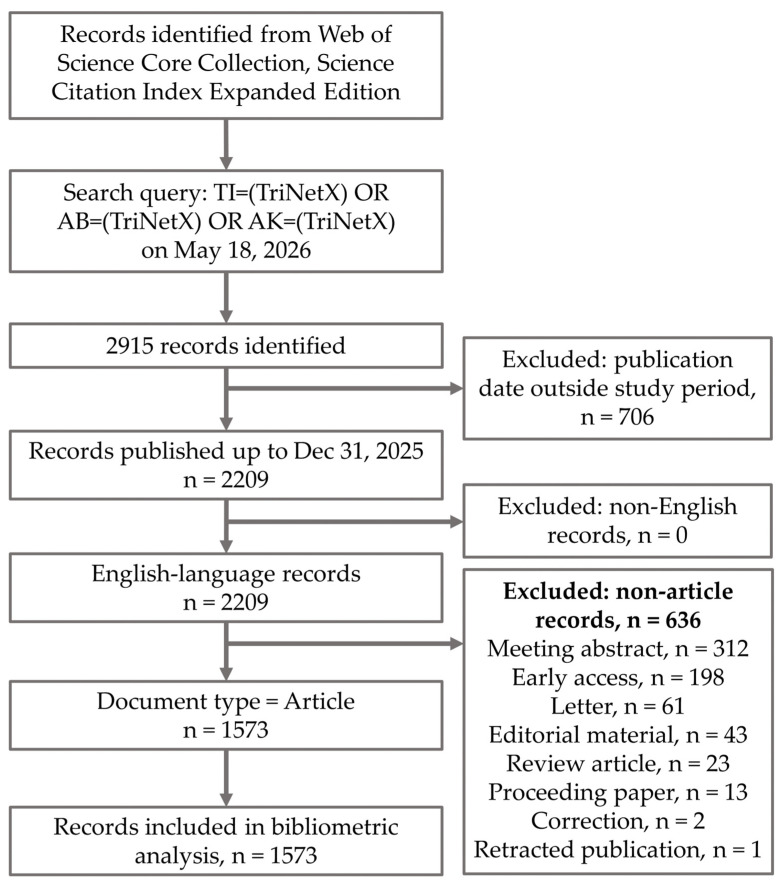
Flow diagram of record identification and selection for the bibliometric analysis. The restriction to Web of Science document type “Article” excluded 636 unique non-article records. The document-type labels listed for excluded records are Web of Science labels and are not mutually exclusive; therefore, their counts sum to 653 and should not be added to derive the number of unique excluded records.

The record identification and selection process is shown in Figure 1. Records were restricted to publications indexed through 31 December 2025, leaving 2209 records within the study period. No records were excluded on the basis of language because all eligible records were in English. The dataset was then limited to WoS document type “Article,” consistent with the study objective of mapping primary real-world evidence studies generated using TriNetX. Other document types, including meeting abstracts, early access records, letters, editorial materials, review articles, proceeding papers, corrections, and retracted publications, were excluded because they did not represent primary data-generating studies using the network. After applying these criteria, 1573 articles were retained for the bibliometric analysis.

To assess potential false-positive inclusion, all 1573 included records were screened for the presence of “TriNetX” in the available WoS bibliographic fields used for corpus identification, including article title, abstract, and author keywords. Records without “TriNetX” in these fields were prespecified for manual review as potentially ambiguous inclusions. In addition, a year-stratified 10% random sample was manually reviewed. This manual review assessed whether TriNetX was used as a data source, analytic platform, cohort-identification tool, or real-world evidence network in the reported study, rather than being mentioned only incidentally.

### 2.2. Bibliometric Analysis

All bibliometric analyses were conducted in R programming environment (version 4.4.0) using the bibliometrix package (version 5.1.0) [16]. The Biblioshiny (version 5.0) web interface was used for generating trend topic analysis. VOSviewer (version 1.6.20) was used for author-keyword co-occurrence mapping [17].

A logistic growth model was fitted to the cumulative annual publication data using the life cycle function implemented in Biblioshiny: P(t) = K/(1 + exp(−b(t − t_0_))), where P(t) is the cumulative number of publications at time t, K is the saturation ceiling, b is the growth rate parameter, and t_0_ is the inflection point. Two key model parameters were estimated: the saturation ceiling (K) and the peak year (T_m_), at which annual output is projected to be highest. Model fit was assessed using R^2^ and root mean square error (RMSE).

#### 2.2.1. Country and Regional Level Productivity Analysis

Country and regional productivity was analyzed using a custom R script, rather than the default Bibliometrix country extraction routine, to normalize country name variants and to preserve the regional distinctions of Taiwan, Hong Kong, and Macau, which are absorbed into China when WoS records list affiliations under “Peoples R China.” Country attribution was based on the corresponding-author affiliation parsed from the reprint author (RP) field of each WoS record. For RP entries assigned to China, Taiwan, Hong Kong, and Macau were identified by pattern matching against the full affiliation text, using curated lists of city names, university names, and hospital names specific to each region. For each paper with N corresponding authors, a fractional weight of 1/N was assigned to each corresponding author so that the total weight per paper summed to 1.0, and these author-level weights were then aggregated by country. The complete script, including the full pattern lists and disambiguation logic, is provided in Supplementary File S1.

#### 2.2.2. Institutional Productivity Analysis

Institutional productivity was assessed using article-level full counting based on WoS organization-enhanced affiliation labels, without manual consolidation of parent systems, campuses, hospitals, or health systems. For each article, duplicate occurrences of the same institution were removed, so that an institution received one credit per article regardless of the number of affiliated authors. Institutional counts therefore represent the number of articles in which each institution appeared, rather than fractional shares of article output. This approach reduced inflation from multiple coauthors within the same institution while avoiding subjective fractional allocation or manual merging of institutional entities.

#### 2.2.3. Journal Productivity and Citation Impact Analysis

Journals were ranked by number of included articles. For each journal, total citation count, 2024 Journal Impact Factor (JIF), Journal Citation Reports (JCR) category, and JIF quartile were recorded.

Citation counts were extracted from WoS on 18 May 2026. To account for differences in publication age, mean citations per year were calculated as total citations divided by the number of years from publication year to 2026, inclusive.

#### 2.2.4. Keyword Harmonization and Co-Occurrence Network Analysis

Author keywords were harmonized before co-occurrence network analysis using a custom VOSviewer thesaurus to improve interpretability. The thesaurus included merge rules for hyphenation and punctuation variants, acronyms and expanded forms, and lexical synonyms. For example, variants of glucagon-like peptide-1 receptor agonists (GLP-1 RA) were consolidated under a single canonical label, and terms related to severe acute respiratory syndrome coronavirus-2 (SARS-CoV-2) and coronavirus disease 2019 were merged under “COVID-19.” Clinically distinct concepts, including type 1 and type 2 diabetes and individual drug names within the same therapeutic class, were retained as separate terms.

Generic terms were excluded when they provided limited thematic specificity. These included platform-related terms, study-design descriptors, broad outcome or exposure terms, and demographic or geographic descriptors. Terms appearing only once in the corpus were not considered for thesaurus construction because they could not independently meet the minimum occurrence threshold. For network readability, well-established and clinically unambiguous abbreviations were used as canonical labels after first definition, including acute kidney injury (AKI), chronic kidney disease (CKD), inflammatory bowel disease (IBD), GLP-1 RA, immune checkpoint inhibitor (ICI), metabolic dysfunction-associated steatotic liver disease (MASLD), and sodium–glucose cotransporter 2 inhibitors (SGLT2i). The VOSviewer thesaurus is provided in Supplementary File S2.

Because the clustering resolution parameter in VOSviewer affects cluster granularity, sensitivity checks were conducted using resolution values of 0.5, 0.7, 1.0, and 1.2. The main thematic structure was stable across these settings. A resolution value of 0.7 was selected for the final visualization because it produced clinically interpretable clusters while avoiding excessive merging at lower resolution and over-fragmentation at higher resolution.

#### 2.2.5. Trend-Topic Analysis

Trend-topic analysis was conducted in Biblioshiny using harmonized author keywords. To improve consistency with the co-occurrence network analysis, the corresponding keyword harmonization files were applied, including a stopword list for generic terms and a synonyms file for variant keyword forms. Keywords were positioned along the temporal axis according to the median publication year of the articles in which they appeared, while horizontal bars represented the interquartile range. The analysis used a minimum keyword frequency of five, with a maximum of five keywords displayed per year.

#### 2.2.6. Clinical Domain–Study Purpose Mapping Method

Clinical domain–study purpose mapping was generated using a reproducible, rule-based dictionary implemented in R (Supplementary File S3). A rule-based approach was selected because it allowed each assignment to be traced to explicit terms and matching rules, making the classification process transparent, inspectable, reproducible, and adaptable by other researchers. The dictionary was therefore intended as a structured classification framework rather than as a fixed universal taxonomy.

For clinical-domain assignment, the classifier used article titles and author keywords, with a title-first rule and author keywords used to support classification when title information was insufficient. For study-purpose assignment, the classifier used article titles, abstracts, and author keywords.

For the domain-level and domain–purpose matrix analyses, fractional weighting was used to avoid double counting. After rare-domain collapsing for the matrix, each article contributed a total weight of 1.0 across its assigned clinical-domain and study-purpose combinations. Specifically, when an article was assigned to D matrix-level clinical domains and P study-purpose labels, each domain–purpose cell received a weight of 1/(D × P). Purpose labels classified as “Other” were excluded from the main domain–purpose matrix because the matrix focused on the four prespecified study-purpose categories.

Because the initial dictionary produced several recognizable error patterns, an iterative audit-and-refinement procedure was applied before generating the final domain–purpose matrix. Misclassification patterns were traced back to the dictionary rules and used to revise the R code. Revisions included adding missing specialty-specific terms, retaining clinically meaningful low-frequency domains for audit purposes, separating article-level labels from matrix-level collapsed labels, adding override rules to reduce false-positive assignments, and adding word-boundary anchors to terms that could otherwise match substrings within unrelated words. For example, boundary control was added to avoid matching “urolog” within “neurological,” “bladder” within “gallbladder,” and “vision” within “revision,” “provision,” or “division.” After these refinements, the corrected classifier was rerun for the full WoS corpus of 1573 records, and the final clinical domain–study purpose matrix was generated from the full corrected dataset.

To assess classification reliability, a year-stratified random audit of 162 records was conducted. The sample was drawn by stratifying the full WoS corpus by publication year and randomly selecting records within each year stratum, with annual stratum sizes rounded up. Two human auditors independently reviewed each sampled record using the article title and, when necessary, the abstract. Clinical-domain labels and study-purpose labels were audited separately. For each dimension, the assigned label set was rated as “accurate” if the classification matched the article cleanly, “fine” if the classification was defensible despite minor over-labeling or under-labeling, or “incorrect” if it contained a clearly spurious label or missed a primary domain or purpose.

Interrater agreement was assessed using observed agreement and unweighted Cohen’s kappa. Because “accurate” and “fine” both represented usable classifications for descriptive mapping, a secondary binary assessment combined these two categories as “acceptable” and compared them with “incorrect.”

#### 2.2.7. Cross-Database Validation Using Scopus

To assess whether the WoS-based findings were dependent on database coverage, a supplementary validation analysis was conducted in Scopus. An equivalent title–abstract–author keyword search was performed using the query: (TITLE(“TriNetX”) OR ABS(“TriNetX”) OR AUTHKEY(“TriNetX”)) AND PUBYEAR < 2026 AND LANGUAGE(english) AND DOCTYPE(ar). This strategy was designed to broadly reproduce the WoS title–abstract–author keyword search while applying the same publication-year, language, and document-type restrictions. The Scopus dataset was analyzed separately and was not merged with the WoS corpus.

Validation focused on pattern-level reproducibility rather than direct numerical replication, given known differences between WoS and Scopus in journal coverage, indexing practices, citation records, and affiliation metadata. The two datasets were compared descriptively in terms of annual publication trends, leading publication sources, and the distribution of articles across the clinical domain and study-purpose classification scheme.

Annual article counts and citation patterns were also compared between the WoS and Scopus datasets. Spearman’s rank correlation coefficient (ρ) was calculated across publication years to assess cross-database concordance in annual publication output and citation patterns.

Publication sources were ranked separately in WoS and Scopus according to the number of included articles. Because several sources had identical article counts, tied ranks were retained, resulting in more than 10 journal titles within the top 10 rank positions in each database. Source-level concordance was assessed by calculating the number and percentage of overlapping journals among all sources included in these rank positions. Jaccard similarity was calculated as the number of shared journals divided by the number of unique journals appearing in either list.

To evaluate whether the clinical domain–study purpose mapping findings were sensitive to WoS coverage, the same rule-based classification dictionary developed for the WoS corpus was applied to the Scopus records without modification. Clinical domains were identified using article titles and author keywords, whereas study purpose categories were identified using article titles, abstracts, and author keywords. Articles assigned to multiple clinical domains were handled using fractional weighting to avoid double counting in the domain-level and domain–purpose matrices. The Scopus-derived clinical domain × study purpose matrix was then compared with the WoS matrix at both the overall clinical-domain level and the domain–purpose cell level. Concordance was assessed descriptively using overlap of top-ranked domains and domain–purpose cells, the number of shared nonzero cells, Jaccard similarity, and Spearman’s rank correlation for proportional distributions. These analyses were interpreted as pattern-level sensitivity checks rather than formal tests of equivalence between databases.

## 3. Results

### 3.1. Dataset Overview and Publication Trends

The final analytic corpus comprised 1573 articles published between 2018 and 2025. False-positive inclusion screening showed that all 1573 records contained “TriNetX” in at least one checked WoS bibliographic field, and no metadata-level false-positive records were identified. In the year-stratified 10% random validation sample, with annual stratum sizes rounded up, all 162 records were confirmed to use TriNetX in the reported study rather than mentioning it only incidentally. The articles were contributed by 5726 authors and indexed in 548 journals. The mean number of coauthors was 6.7 per article.

**Figure 2:**
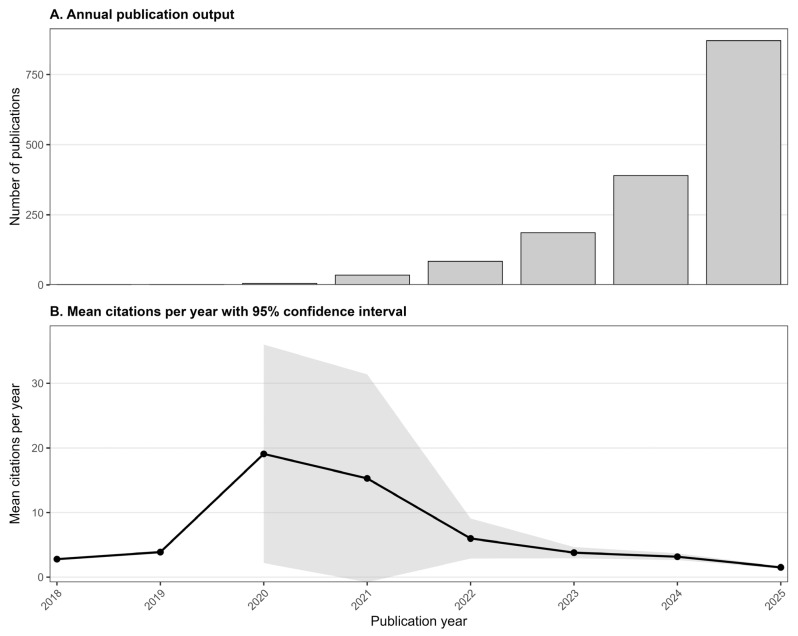
Annual article counts (**A**) and mean citations per year of TriNetX-based articles (**B**), 2018–2025. The shaded area in Panel (**B**) represents the 95% confidence interval around the mean number of citations per year.

TriNetX-related output grew slowly at first, then rose steeply in annual publications from the early 2020s onward. Annual article counts increased from 1 publication in 2018 to 871 publications in 2025. A logistic growth model was fitted to the cumulative publication curve as a descriptive summary of the observed growth pattern. The model closely followed the cumulative data over the study period, with an R^2^ of 0.99 and an RMSE of 6.89. However, because the model was based on only eight annual observations and most of the increase occurred in the final years of the study period, the projected peak around 2028 should be interpreted as an exploratory indication of possible deceleration rather than a validated forecast of future publication output (Figure 2).

### 3.2. Country and Regional Productivity

Country and regional productivity was highly concentrated among a small number of contributors (Table 1). Based on fractional counting of corresponding-author affiliations, the United States ranked first with 982.4 articles, accounting for 62.5% of the total fractional output. Taiwan ranked second with 336.4 articles (21.4%), followed by the United Kingdom with 70.7 articles (4.5%) and Germany with 48.8 articles (3.1%). Other countries contributed smaller proportions, including Italy, Israel, Brazil, France, China, Japan, and Spain, each accounting for less than 1% of the total fractional output. In terms of international collaboration, Taiwan had the highest proportion of multiple-country publications among the leading contributors (37.4%), followed by the United States (16.4%) and the United Kingdom (12.7%). Citation patterns differed from publication volume. Although the United States accumulated the largest total fractional citation count (7686.1), the United Kingdom showed the highest mean article citations (57.5) and the highest mean citations per year (15.0 per year), suggesting greater citation impact relative to its publication volume.

### 3.3. Institutional Productivity

Institutional productivity was concentrated in several major research hubs in the United States and Taiwan (Table 2). The University System of Ohio ranked first, appearing in 180 articles (11.4%), followed by Chi Mei Hospital in Taiwan with 165 articles (10.5%), Case Western Reserve University with 162 articles (10.3%), and the University of Texas System with 161 articles (10.2%). Other leading institutions included Chung Shan Medical University (153 articles, 9.7%), the Pennsylvania Commonwealth System of Higher Education (144 articles, 9.2%), Chung Shan Medical University Hospital (138 articles, 8.8%), Pennsylvania State University (125 articles, 7.9%), Penn State Health (120 articles, 7.6%), and the University of Texas Medical Branch Galveston (115 articles, 7.3%). Citation indicators varied across institutions. The University System of Ohio had the highest total citation count among the leading institutions (1417 citations), whereas Chung Shan Medical University Hospital had the highest citations per article (8.9). Chi Mei Hospital had the highest mean citations per year (4.8 citations per year), followed by Case Western Reserve University, Chung Shan Medical University, and Chung Shan Medical University Hospital, each with 4.4 citations per year. TriNetX-based research output was led mainly by large United States university systems and health systems, with notable contributions from Taiwanese medical centers and universities.

### 3.4. Journal Productivity

TriNetX-based studies were published across a diverse range of clinical and multidisciplinary journals, with no single journal dominating the publication landscape (Table 3). Among the leading sources, the *Journal of Clinical Medicine* ranked first with 32 articles (2.0%), followed by the *Journal of Medical Virology* with 23 articles (1.5%)*. JAMA Network Open* and *Scientific Reports* ranked third, each publishing 21 articles (1.3%), while *Diabetes Research and Clinical Practice* ranked fourth with 20 articles (1.3%). Several journals shared fifth place with 19 articles each (1.2%), including *Frontiers in Nutrition*, *International Journal of Pediatric Otorhinolaryngology*, *Laryngoscope*, and *Otolaryngology–Head and Neck Surgery*. The remaining journals within the top 10 rank positions included *Diabetes, Obesity and Metabolism*, *American Journal of Ophthalmology*, *Urology*, *PLoS One*, *International Journal of Medical Sciences*, and *Obesity Surgery*. Most leading journals were ranked in the first quartile of their respective WoS categories, including journals in general medicine, virology, endocrinology and metabolism, nutrition and dietetics, ophthalmology, surgery, and multidisciplinary sciences. In short, TriNetX-based research has reached a broad range of specialty and general biomedical journals, with particularly visible representation in general medicine, virology, endocrinology and metabolism, otorhinolaryngology, and multidisciplinary titles.

### 3.5. Most-Cited Articles

The most-cited TriNetX-related articles were largely concentrated in COVID-19 research, particularly studies examining neurological, psychiatric, cardiovascular, autoimmune, and treatment-related outcomes (Table 4). The three highest-cited articles were all led by Taquet and published in *Lancet Psychiatry*. The leading article, which examined 6-month neurological and psychiatric outcomes among 236,379 COVID-19 survivors, received 1466 citations and had the highest mean citation rate at 244.3 citations per year [18]. The second and third most-cited articles also focused on psychiatric or neurological outcomes after severe acute respiratory syndrome coronavirus-2 (SARS-CoV-2) infection, receiving 890 and 565 citations, respectively [19,20].

Beyond these neuropsychiatric studies, highly cited TriNetX-related articles addressed long-term cardiovascular outcomes after COVID-19 [21], case-fatality trends among individuals with intellectual and developmental disabilities [22], autoimmune disease risk after COVID-19 [23], breakthrough infections [24], neuropsychiatric manifestations [25], and nirmatrelvir–ritonavir treatment [26]. One non-COVID-19 article, published in *Nature Medicine*, examined the association between semaglutide and suicidal ideation in a real-world cohort and ranked seventh [27]. On the whole, the citation pattern indicates that early high-impact TriNetX-related research focused on urgent COVID-19 questions, while more recent highly cited work has begun to extend into pharmacovigilance and real-world safety topics.

**Table 4:** Top 10 most-cited TriNetX-based articles indexed in Web of Science, 2018–2025.

Rank	First Author (Year of Publication) [Reference]	Article Title	Journal (2024 JIF)	Total Citations	Mean Citations per Year
1	Taquet, Maxime (2021) [18]	6-month neurological and psychiatric outcomes in 236 379 survivors of COVID-19: A retrospective cohort study using electronic health records	*Lancet Psychiatry* (24.8)	1466	244.3
2	Taquet, Maxime (2021) [19]	Bidirectional associations between COVID-19 and psychiatric disorder: Retrospective cohort studies of 62 354 COVID-19 cases in the USA	*Lancet Psychiatry* (24.8)	890	148.3
3	Taquet, Maxime (2022) [20]	Neurological and psychiatric risk trajectories after SARS-CoV-2 infection: An analysis of 2-year retrospective cohort studies including 1 284 437 patients	*Lancet Psychiatry* (24.8)	565	113.0
4	Wang, Weijie (2022) [21]	Long-term cardiovascular outcomes in COVID-19 survivors among non-vaccinated population: a retrospective cohort study from the TriNetX US collaborative networks	*EClinicalMedicine* (10.0)	275	55.0
5	Turk, Margaret A (2020) [22]	Intellectual and developmental disability and COVID-19 case-fatality trends: TriNetX analysis	*Disability and Health Journal* (3.3)	270	38.6
6	Chang, Renin (2023) [23]	Risk of autoimmune diseases in patients with COVID-19: A retrospective cohort study	*EClinicalMedicine* (10.0)	237	59.2
7	Wang, William (2024) [27]	Association of semaglutide with risk of suicidal ideation in a real-world cohort	*Nature Medicine* (50.0)	195	65.0
8	Taquet, Maxime (2022) [24]	Six-month sequelae of post-vaccination SARS-CoV-2 infection: A retrospective cohort study of 10,024 breakthrough infections	*Brain, Behavior, and Immunity* (7.6)	180	36.0
9	Nalleballe, Krishna (2020) [25]	Spectrum of neuropsychiatric manifestations in COVID-19	*Brain, Behavior, and Immunity* (7.6)	172	24.6
10	Ganatra, Sarju (2023) [26]	Oral nirmatrelvir and ritonavir in nonhospitalized vaccinated patients with coronavirus disease 2019	*Clinical Infectious Diseases* (7.3)	119	29.8

JIF: Journal Impact Factor. Mean citations per year were calculated as total citations divided by the number of years from publication year to 2026, inclusive.

### 3.6. Author-Keyword Co-Occurrence Network Analysis

**Figure 3:**
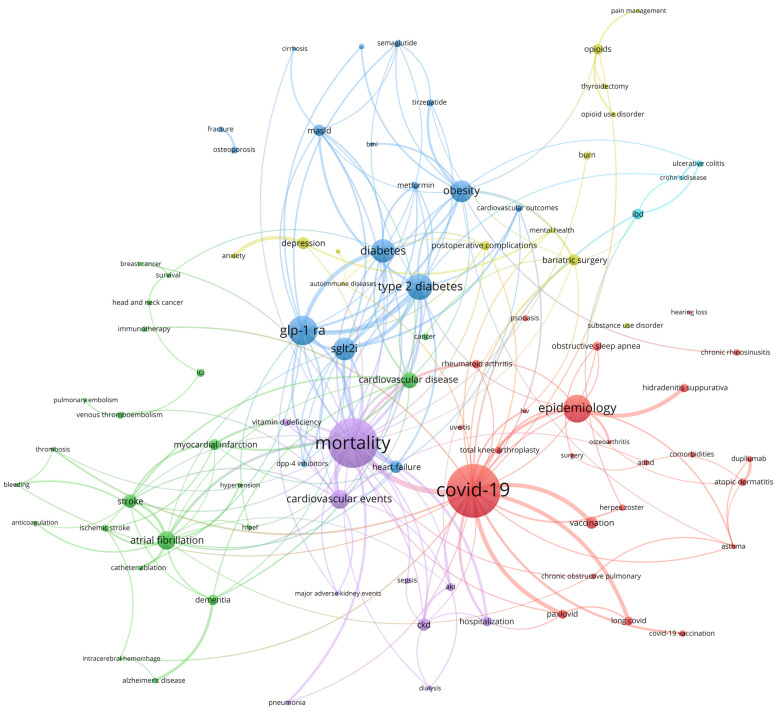
Author-keyword co-occurrence network of TriNetX-based articles, 2018–2025. Nodes represent harmonized author keywords, and larger nodes indicate more frequent keywords. Links represent keyword co-occurrence within the same article, with closer positioning indicating stronger relatedness. Colors indicate clusters of keywords that frequently co-occur and therefore represent major thematic areas in the TriNetX publication corpus.

The author-keyword co-occurrence network identified six color-coded clusters, indicating that TriNetX-based studies were organized around several clinically distinct research areas (Figure 3). The red cluster was centered on COVID-19 and epidemiology, with related terms including vaccination, Paxlovid, long COVID, herpes zoster, chronic obstructive pulmonary disease (COPD), asthma, atopic dermatitis, hidradenitis suppurativa, and rheumatoid arthritis. The blue cluster was dominated by metabolic and cardiometabolic topics, particularly GLP-1 RA, type 2 diabetes, diabetes, SGLT2i, obesity, MASLD, metformin, semaglutide, tirzepatide, and dipeptidyl peptidase-4 (DPP-4) inhibitors. The purple cluster was centered on outcome-oriented terms, especially mortality, which was the largest node in this cluster, together with cardiovascular events, CKD, hospitalization, AKI, dialysis, sepsis, pneumonia, and major adverse kidney events.

The green cluster grouped cardiovascular, cerebrovascular, thromboembolic, oncologic, and neurodegenerative topics, including atrial fibrillation, cardiovascular disease, stroke, myocardial infarction, ischemic stroke, venous thromboembolism, dementia, Alzheimer’s disease, cancer, ICI, and immunotherapy. The yellow cluster included mental health, surgical, and pain- or opioid-related topics, with prominent terms such as depression, bariatric surgery, opioids, postoperative complications, anxiety, mental health, substance use disorder, opioid use disorder, burn, and thyroidectomy. A smaller cyan cluster represented inflammatory bowel disease research and consisted of IBD, ulcerative colitis, and Crohn’s disease. On the whole, the most prominent author keywords were COVID-19, mortality, GLP-1 RA, type 2 diabetes, diabetes, SGLT2i, obesity, and epidemiology, with cross-cluster connections between metabolic, cardiovascular, infectious disease, and outcome-related topics.

### 3.7. Author-Keyword Trend-Topic Analysis

**Figure 4:**
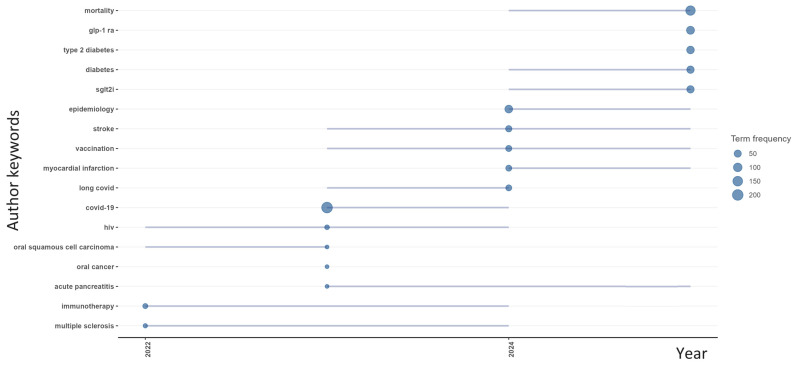
Trend-topic analysis of author keywords in TriNetX-based articles, 2018–2025.

The trend-topic analysis identified 17 author keywords with distinct temporal patterns (Figure 4). Earlier trend terms were mainly concentrated around 2022–2023, including “immunotherapy” (frequency = 11; median year = 2022), “multiple sclerosis” (frequency = 6; median year = 2022), “human immunodeficiency virus” (HIV; frequency = 8; median year = 2023), “oral squamous cell carcinoma” (frequency = 5; median year = 2023), “oral cancer” (frequency = 5; median year = 2023), and “acute pancreatitis” (frequency = 5; median year = 2023). “COVID-19” was the most frequent trend topic overall (frequency = 205), with a median year of 2023 and an interquartile range extending from 2023 to 2024, indicating that COVID-19-related TriNetX studies were especially prominent during this period.

From 2024 onward, the trend topics shifted toward epidemiological, cardiovascular, post-COVID, and metabolic themes. Terms with a median year of 2024 included “epidemiology” (frequency = 70), “stroke” (frequency = 30), “vaccination” (frequency = 26), “long COVID” (frequency = 24), and “myocardial infarction” (frequency = 24). The most recent trend terms were concentrated in 2025, particularly “mortality” (frequency = 138), “GLP-1 RA” (frequency = 77), “type 2 diabetes” (frequency = 65), “diabetes” (frequency = 57), and “SGLT2i” (frequency = 56). These findings show that recent author-keyword trends in TriNetX-based studies have moved from pandemic-related topics toward clinical outcomes, cardiometabolic diseases, and medication-related themes.

### 3.8. Clinical Domain–Study Purpose Mapping

**Figure 5:**
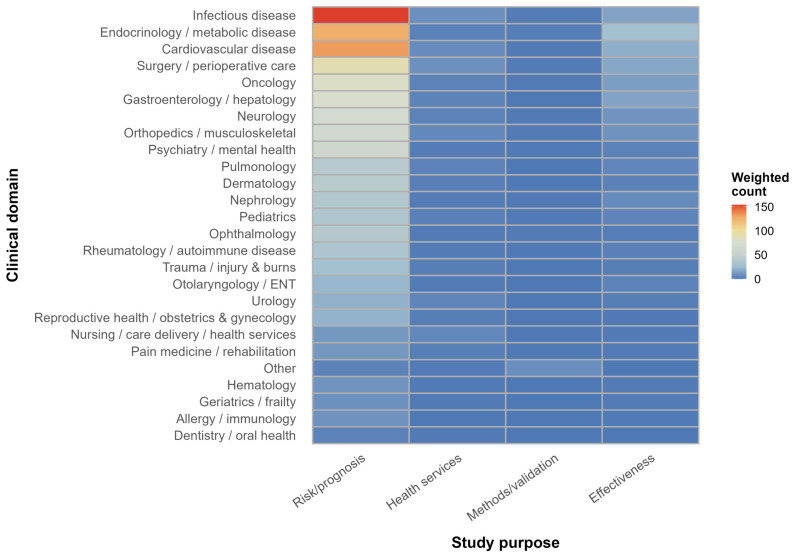
Clinical domain–study purpose mapping of TriNetX-based articles, 2018–2025. Rows represent clinical domains, and columns represent study-purpose categories. Cell intensity represents the fractional weighted number of articles assigned to each clinical domain–study purpose combination, with darker cells indicating greater publication concentration. Clinical domains are ranked by total weighted count. Fractional weighting was used so that articles assigned to multiple domains or purposes contributed a total weight of 1.0 across their assigned combinations.

The clinical domain–study purpose heatmap showed that TriNetX-based studies were distributed across a wide range of clinical areas, but the distribution was uneven across both domains and study purposes (Figure 5). Risk/prognosis was the dominant purpose category, with the strongest concentrations observed in infectious disease, endocrinology/metabolic disease, cardiovascular disease, surgery/perioperative care, oncology, gastroenterology/hepatology, orthopedics/musculoskeletal disorders, neurology, and psychiatry/mental health. In contrast, studies categorized as health services, methods/validation, and effectiveness were less frequent across most domains. Effectiveness-oriented studies were present but comparatively limited, suggesting that much of the TriNetX literature has focused on risk estimation, prognosis, outcome prediction, and epidemiologic associations rather than direct evaluation of interventions or comparative treatment effects.

Several clinical areas appeared sparsely represented in the matrix, including geriatrics/frailty, allergy/immunology, dentistry/oral health, hematology, pain medicine/rehabilitation, and reproductive health/obstetrics and gynecology. These lower-frequency domains should be interpreted cautiously because some categories were retained for clinical interpretability despite small counts.

The reliability audit of the revised rule-based classifier showed high agreement between the two human auditors. For clinical-domain classification, initial agreement was 96.3% across the three verdict categories, with an unweighted Cohen’s kappa of 0.92 (bootstrap 95% CI, 0.87–0.98). When “accurate” and “fine” were combined as acceptable classifications and compared with “incorrect,” agreement was 100.0% and Cohen’s kappa was 1.00. The six domain disagreements were all between “accurate” and “fine,” and none involved the “incorrect” category.

For study-purpose classification, initial agreement was 87.0% across the three verdict categories, with an unweighted Cohen’s kappa of 0.72 (bootstrap 95% CI, 0.60–0.82). When “accurate” and “fine” were combined as acceptable classifications and compared with “incorrect,” agreement was 98.1% and Cohen’s kappa was 0.89 (bootstrap 95% CI, 0.75–1.00). Of the 21 purpose disagreements, 18 were between “accurate” and “fine,” whereas only three crossed the acceptability boundary between acceptable and incorrect classification. These findings indicate that the revised rule-based classifier was sufficiently reliable for descriptive domain–purpose mapping, while also showing that study-purpose classification was more judgment-dependent than clinical-domain classification.

### 3.9. Scopus Validation of WoS-Derived Bibliometric and Clinical Domain–Study Purpose Mapping

The Scopus validation analysis reproduced the rapid recent expansion of TriNetX-related publications observed in the WoS dataset (Supplementary Figure S1). Annual output was minimal before 2021, increased steadily from 2021 onward, and rose sharply in 2024 and 2025, with 2025 accounting for the largest number of Scopus-indexed publications. Annual publication counts showed perfect rank-order concordance between WoS and Scopus across 2018–2025 (Spearman’s ρ = 1.00), reflecting the same monotonic growth pattern in both databases. This statistic was interpreted descriptively because it was based on only eight yearly observations and was strongly influenced by the shared upward temporal trend. The citation indicator showed higher mean citations per year among earlier publications, particularly those published in 2020 and 2021, followed by lower values in more recent years. The confidence intervals were wide for earlier years, reflecting the small number of publications in those periods.

The leading publication sources were also broadly consistent between WoS and Scopus (Supplementary Table S1). *Journal of Clinical Medicine* ranked first in WoS and second in Scopus, with 32 articles in both databases. Several other high-frequency sources appeared in both rankings, including *Journal of Medical Virology*, *JAMA Network Open*, *Scientific Reports*, *Diabetes Research and Clinical Practice*, *Frontiers in Nutrition*, *International Journal of Pediatric Otorhinolaryngology*, *Laryngoscope*, *American Journal of Ophthalmology*, *Urology*, *Obesity Surgery*, and *International Journal of Medical Sciences*. After harmonizing minor source-title variants, 14 of the 15 WoS top-ranked sources also appeared among the Scopus top-ranked sources, whereas 14 of the 16 Scopus top-ranked sources also appeared in the WoS list. The Jaccard similarity coefficient was 0.824, indicating substantial overlap in the publication-source structure across databases. The main database-specific differences were the appearance of *Journal of the American Academy of Dermatology* and *Journal of Clinical Oncology* among the Scopus leading sources, whereas *PLoS One* appeared only in the WoS top-ranked list. These differences likely reflect database-specific journal coverage, indexing practices, and source-title formatting rather than a substantive divergence in publication patterns.

The Scopus validation analysis further showed that the clinical domain-study purpose pattern observed in WoS was broadly reproduced in an independent database (Supplementary Figure S2). At the overall clinical-domain level, the distribution was highly concordant between WoS and Scopus. Infectious disease ranked first in both databases, accounting for 11.9% of the weighted domain distribution in WoS and 10.7% in Scopus. In Scopus, the remaining top 10 domains, in descending order, were cardiovascular disease, endocrinology/metabolic disease, surgery/perioperative care, orthopedics/musculoskeletal disorders, oncology, gastroenterology/hepatology, neurology, psychiatry/mental health, and dermatology. All of these domains also appeared among the top-ranked domains in WoS, supporting broad cross-database concordance. Overall clinical-domain proportions showed high rank-order concordance between WoS and Scopus (Spearman’s ρ = 0.988), indicating that the relative ordering of clinical domains was highly similar across databases.

At the domain–purpose cell level, 93 shared nonzero cells were identified among 95 unique nonzero cells across the two databases, corresponding to a Jaccard similarity of 0.979. Rank-order concordance was high for domain–purpose cell proportions (Spearman’s ρ = 0.977). The top 10 and top 20 domain–purpose cells were identical across WoS and Scopus, and 28 of the top 30 cells overlapped.

## 4. Discussion

### 4.1. Rapid Expansion and Maturation of TriNetX-Based Research

This bibliometric analysis of 1573 Web of Science-indexed original articles shows that TriNetX-based research has moved from early adoption to rapid expansion. Annual output increased from 1 article in 2018 to 871 articles in 2025, indicating that the platform has become a widely used infrastructure for real-world evidence generation across clinical medicine. The logistic growth model should therefore be interpreted as exploratory. Although the model suggested a possible peak around 2028, this estimate was derived from only eight annual observations and may be sensitive to short-term changes in indexing, database access, institutional licensing, journal publication practices, competing federated data networks, and research priorities. The finding is best viewed as a descriptive signal of possible growth deceleration rather than as a reliable forecast of future TriNetX-related publication output.

The observed growth is consistent with the broader demand for large-scale, analysis-ready electronic health record data [28]. Early highly cited TriNetX studies were concentrated in COVID-19 research, reflecting the platform’s ability to support rapid analyses during a public health emergency. More recent trends show expansion into chronic disease, cardiometabolic medicine, pharmacoepidemiology, and long-term outcomes. This shift suggests that TriNetX is no longer used mainly as a pandemic-response data source, but has become part of the routine evidence-generation infrastructure for observational clinical research.

### 4.2. Geographic Concentration and Institutional Research Hubs

The geographic distribution of publications was highly concentrated. The United States accounted for the largest share of fractional corresponding-author output, followed by Taiwan and the United Kingdom. The predominance of the United States may partly reflect the historical development and early institutional adoption of TriNetX within the United States healthcare and academic systems, as well as the concentration of research-intensive institutions with access to the platform. Taiwan’s strong contribution is also notable. Rather than reflecting population size, this pattern may be related to concentrated institutional access, active research teams, and Taiwan’s established experience in secondary data research, including prior use of the NHIRD [29].

These country-level patterns should be interpreted cautiously. Publication volume does not necessarily indicate scientific leadership alone, because productivity may also be shaped by differential access to TriNetX, licensing arrangements, analytical capacity, institutional publication cultures, journal indexing patterns, and affiliation-reporting practices. Therefore, the geographic findings are best interpreted as indicators of publication concentration within WoS-indexed TriNetX-related literature, rather than as definitive evidence of national dominance in real-world evidence research.

The institutional findings point in the same direction. Leading contributors were concentrated in several research hubs, including major United States university systems and health systems, as well as Taiwanese institutions such as Chi Mei Hospital, Chung Shan Medical University, and Chung Shan Medical University Hospital. This pattern is understandable because analytic teams often develop within local health systems and academic networks. However, it also suggests that, despite TriNetX’s federated design, the published literature captured in WoS may still be shaped more by concentrated institutional access and established analytic capacity than by broadly distributed cross-regional research participation.

### 4.3. Publication Venues and Citation Influence

TriNetX-based studies were disseminated across several hundred journals, with leading sources spanning general medicine, virology, endocrinology, nutrition, otorhinolaryngology, ophthalmology, urology, surgery, and multidisciplinary science. No single journal dominated the publication landscape, suggesting that TriNetX has been adopted across many clinical specialties rather than being confined to a narrow informatics or real-world data literature. The appearance of multiple first-quartile journals among the leading sources also indicates that studies using TriNetX data can reach clinically influential publication venues when the research question, cohort definition, and analytic design are compelling.

Citation patterns should be interpreted in relation to timing and topic. The most-cited articles were dominated by COVID-19 studies, especially those addressing neurological, psychiatric, cardiovascular, autoimmune, and treatment-related outcomes. This concentration reflects the exceptional scientific and public health attention given to COVID-19, rather than citation influence being evenly distributed across all TriNetX-based research. The presence of a highly cited non-COVID-19 pharmacovigilance study on semaglutide [27] suggests that the platform is also gaining visibility in medication safety and comparative risk assessment. Future citation patterns may therefore become less pandemic-centered as more studies examine chronic disease management, drug safety, and long-term outcomes.

### 4.4. Thematic Evolution from COVID-19 to Cardiometabolic and Outcomes Research

The author-keyword co-occurrence network showed that TriNetX-based research is organized around several clinically recognizable themes. COVID-19 and infectious disease remained a major cluster, but cardiometabolic and outcome-oriented topics were also prominent. Keywords related to GLP-1 RA, SGLT2i, type 2 diabetes, obesity, CKD, cardiovascular events, hospitalization, and mortality suggest increasing use of TriNetX for studying chronic disease trajectories and medication-related outcomes.

The trend-topic analysis provides additional temporal context. Earlier topics were more closely linked to COVID-19, immunotherapy, and selected specialty-specific conditions, whereas the most recent terms were concentrated around mortality, type 2 diabetes, GLP-1 RA and SGLT2i. This pattern suggests a transition from urgent pandemic-related analyses toward questions involving long-term prognosis, cardiometabolic care, and treatment safety. However, keyword-based thematic analysis depends on author terminology, indexing practices, and the applied thesaurus. The clusters should therefore be interpreted as a map of visible publication themes, not as a complete measurement of all clinical questions studied using TriNetX.

The transition from COVID-19-centered keywords toward cardiometabolic and outcome-oriented topics may indicate a broader maturation of TriNetX-related research. During the pandemic, federated electronic health record networks supported rapid analyses of infection risk, complications, treatments, and outcomes across large clinical populations. As COVID-19 became less dominant in the literature, TriNetX-related publications increasingly shifted toward chronic disease areas, including diabetes, obesity, cardiovascular disease, renal disease, and multimorbidity, where longitudinal electronic health record data are well suited to studying real-world outcomes. This pattern suggests a movement from event-driven pandemic research toward more sustained use of TriNetX for chronic disease epidemiology, comparative effectiveness, safety assessment, and outcome-risk modeling. However, keyword trends should be interpreted as signals from WoS-indexed articles explicitly mentioning TriNetX, rather than as a complete representation of all research conducted using the platform.

### 4.5. Clinical Domain–Study Purpose Gaps and Implications

The clinical domain–study purpose mapping is the main practice-oriented contribution of this study. Conventional bibliometric indicators show where publications accumulate, who contributes, and which topics cluster together. By contrast, domain–purpose mapping classifies articles according to both the clinical area addressed and the primary purpose of the study. This makes the analysis more directly useful for gap identification because it distinguishes heavily studied clinical fields from heavily studied uses of the platform.

The strongest concentration of studies was observed in risk/prognosis, particularly in infectious disease, endocrinology/metabolic disease, cardiovascular disease, surgery/perioperative care, oncology, gastroenterology/hepatology, orthopedics/musculoskeletal disorders, neurology, and psychiatry/mental health. This concentration is expected because federated EHR data are well suited for estimating incidence, prognosis, comorbidity patterns, and outcome risks in large patient populations.

In contrast, effectiveness-oriented studies, health services research, and methods/validation studies were less frequent across most clinical domains. This imbalance is important. Risk and prognosis studies can identify associations and generate clinically useful hypotheses, but they do not by themselves establish treatment effectiveness, care quality, implementation feasibility, or health-system performance. Where TriNetX data are well suited to the clinical question and required variables are sufficiently captured, future research could place greater emphasis on comparative effectiveness, target-trial emulation [30], long-term safety, diagnostic algorithm validation, phenotype validation, care pathways, treatment sequencing, and disparities in access or outcomes. These directions would move the literature beyond describing risk toward evaluating clinical decisions and health-system practices.

Several domains were sparsely represented, including geriatrics/frailty, allergy/immunology, dentistry/oral health, hematology, pain medicine/rehabilitation, and reproductive health/obstetrics and gynecology. These findings should be interpreted in light of the rule-based classification approach, differences in specialty-specific keyword practices, and the variable suitability of TriNetX data for specific clinical questions. Nevertheless, these lower-frequency areas provide reasonable starting points for future gap-driven research, especially where large sample sizes could help address rare outcomes, understudied populations, multimorbidity, and long-term treatment safety.

For institutions and decision-makers, the findings suggest that database access alone is insufficient. Productivity also depends on organized analytic capacity, repeatable workflows, and methodologically careful study design. Federated EHR networks can support rapid evidence generation, but results should still be interpreted alongside clinical expertise, biological plausibility, randomized evidence when available, and awareness of residual confounding. Large sample size increases precision, but it does not eliminate systematic bias; in fact, it may produce highly precise estimates of a biased effect [31].

A further implication concerns the quality and semantic consistency of the clinical data available for federated EHR research. The value of platforms such as TriNetX depends not only on the number of participating institutions, but also on how completely and consistently clinical information is documented at the point of care. This issue is especially relevant for healthcare professions whose work may be underrepresented in EHR-derived research, including nursing, where assessments, interventions, care coordination, functional status, education, and patient responses are often recorded in less standardized formats. Greater use of structured documentation and standardized terminologies, particularly standardized nursing terminologies, could improve semantic interoperability, reduce information loss across systems, and expand the range of clinical questions that can be studied using federated EHR networks [32]. Future TriNetX-related research may therefore benefit from closer integration between real-world evidence infrastructure, clinical documentation standards, and profession-specific terminologies.

### 4.6. Cross-Database Validation of Web of Science–Derived Patterns

The Scopus validation analysis strengthened confidence that the main findings were not simply artifacts of WoS coverage. Annual publication counts showed identical rank ordering across WoS and Scopus, although this result should be viewed descriptively because it was based on only eight publication years. Leading publication sources also showed substantial overlap across databases, with a Jaccard similarity coefficient of 0.824 after harmonization of source-title variants.

The validation was especially informative for the clinical domain–study purpose mapping. The leading and lowest-frequency clinical domains were highly similar across databases, and the proportional domain rankings showed strong concordance. At the domain-purpose cell level, almost all nonzero cells were shared between the two databases, the top 10 and top 20 cells were identical, and rank-order concordance was high. These results support the main interpretation that TriNetX-based studies are concentrated in risk and prognosis research and that several clinical domains remain comparatively less represented. At the same time, Scopus validation should not be interpreted as proof of numerical equivalence. WoS and Scopus differ in journal coverage, citation indexing, affiliation metadata, and source-title handling, so the validation is best viewed as a pattern-level sensitivity analysis [33].

### 4.7. Strengths and Limitations

This study has several strengths. It provides a comprehensive bibliometric overview of WoS-indexed original TriNetX-based articles published from 2018 to 2025. It used a reproducible search strategy, restricted the corpus to original articles, applied custom country and regional disambiguation, and used article-level institutional counting to reduce inflation from duplicate institutional affiliations within the same paper. The keyword harmonization process reduced redundancy in author-keyword analysis, while the rule-based clinical domain-study purpose mapping extended the analysis beyond conventional bibliometric indicators by identifying where evidence generation is concentrated and where research gaps remain. The Scopus validation analysis further tested whether the main publication and gap-map patterns were dependent on a single bibliographic database, thereby strengthening confidence in the pattern-level interpretation of the results. The agreement of the revised clinical domain–study purpose classifier was further evaluated using a year-stratified random audit by two independent human auditors.

Several limitations should also be considered. First, this was a bibliometric and metadata-based analysis; it did not assess the methodological quality, phenotype validity, causal assumptions, or reproducibility of individual TriNetX studies. Second, the main corpus was restricted to WoS-indexed articles that explicitly mentioned “TriNetX” in the title, abstract, or author keywords. Although this search strategy was reproducible and supported by false-positive inclusion validation, it may have missed articles that used TriNetX without naming the platform in these searchable fields. The findings should therefore be interpreted as a bibliometric map of the search-defined WoS corpus rather than a complete census of all TriNetX-supported studies. Third, although Scopus was used for validation, neither database can capture every relevant publication. Fourth, author-keyword analyses depend on author-supplied terminology and thesaurus decisions. Finally, the clinical domain–study purpose mapping relied on metadata-based rule matching rather than full-text review. Although the revised classifier showed high interrater agreement in a year-stratified two-auditor audit, residual misclassification remains possible, particularly for titles with ambiguous wording, studies with multiple legitimate purposes, and lower-frequency clinical domains.

## 5. Conclusions

This bibliometric analysis of WoS-indexed articles explicitly mentioning TriNetX shows that this publication corpus expanded rapidly from 2018 to 2025 and has become a prominent source of real-world evidence across multiple clinical fields. Publications were concentrated in the United States, Taiwan, and selected institutional research hubs, while author-keyword trends suggested a shift from COVID-19-related studies toward cardiometabolic disease, medication-related outcomes, and prognosis-focused research. The clinical domain-study purpose mapping showed that most studies focused on risk and prognosis, whereas comparative effectiveness, health services, and methods or validation studies were less frequent. Several clinical areas, including geriatrics/frailty, allergy/immunology, dentistry/oral health, hematology, pain medicine/rehabilitation, and reproductive health/obstetrics and gynecology, appeared comparatively less represented in the WoS corpus. The Scopus validation reproduced the main publication patterns and clinical domain–study purpose distributions, indicating that the principal findings were not specific to WoS coverage alone. Where TriNetX data capture, phenotype definitions, and analytic assumptions are suitable for the clinical question, future studies could make greater use of cross-regional collaboration and place more emphasis on clinically actionable questions, including comparative effectiveness, long-term safety, care pathways, and validation of real-world data phenotypes.

## Figures and Tables

**Table 1:** Countries and regions in the top 10 rank positions based on corresponding-author affiliation in TriNetX-based articles, 2018–2025.

Rank	Country	Fractional Article Count (%)	MCP %	Total Citations	Mean Article Citations	Mean Citations per Year
1	United States	982.4 (62.5)	16.4	7686.1	7.8	3.7
2	Taiwan	336.4 (21.4)	37.4	2419.4	7.2	4.3
3	United Kingdom	70.7 (4.5)	12.7	4068.4	57.5	15.0
4	Germany	48.8 (3.1)	3.8	430.1	8.8	4.0
5	Italy	12.6 (0.8)	0.6	37.7	3.0	2.7
6	Israel	10.3 (0.7)	0.3	90.3	8.8	5.0
7	Brazil	9.0 (0.6)	0	47.0	5.2	3.0
8	France	8.7 (0.6)	0.7	21.0	2.4	2.4
9	China	7.9 (0.5)	6.9	25.0	3.2	2.4
10	Japan	7.0 (0.4)	0	24.0	3.4	3.4
10	Spain	7.0 (0.4)	0	77.0	11.0	5.4

MCP: multiple-country publications. Total article production per country/region was calculated from the corresponding author’s country/region of affiliation parsed from the WoS reprint author (RP) field, using a fractional counting approach. Country/region-level fractional citation count was calculated by assigning each corresponding author a fractional share (1/N) of the article’s total citation count, aggregated by country/region.

**Table 2:** Top 10 institutions ranked by article-level full counting in TriNetX-based articles, 2018–2025.

Rank	Institution, Country/Region	Article Count (%)	Total Citations	Citations per Article	Mean Citations per Year
1	University System of Ohio, US	180 (11.4)	1417	7.9	4.1
2	Chi Mei Hospital, Taiwan	165 (10.5)	1201	7.3	4.8
3	Case Western Reserve University, US	162 (10.3)	1365	8.4	4.4
4	University of Texas System, US	161 (10.2)	1048	6.5	3.6
5	Chung Shan Medical University, Taiwan	153 (9.7)	1327	8.7	4.4
6	Pennsylvania Commonwealth System of Higher Education, US	144 (9.2)	786	5.5	2.4
7	Chung Shan Medical University Hospital, Taiwan	138 (8.8)	1230	8.9	4.4
8	Pennsylvania State University, US	125 (7.9)	668	5.3	2.2
9	Penn State Health, US	120 (7.6)	654	5.4	2.3
10	University Of Texas Medical Branch Galveston, US	115 (7.3)	788	6.8	3.6

US: United States. Counts represent the number of articles in which an institution appeared at least once in the affiliation field. Multiple authors from the same institution within a single article contributed one article count.

**Table 3:** Publication sources in the top 10 rank positions for TriNetX-based articles, 2018–2025.

Rank	Journal	Article Count (%)	2024 JIF	JCR Category [JIF Quartile]
1	*Journal of Clinical Medicine*	32 (2.0)	2.9	Medicine, General & Internal [Q1]
2	*Journal of Medical Virology*	23 (1.5)	4.6	Virology [Q1]
3	*JAMA Network Open*	21 (1.3)	9.7	Medicine, General & Internal [Q1]
3	*Scientific Reports*	21 (1.3)	3.9	Multidisciplinary Sciences [Q1]
4	*Diabetes Research and Clinical Practice*	20 (1.3)	7.4	Endocrinology & Metabolism [Q1]
5	*Frontiers in Nutrition*	19 (1.2)	5.1	Nutrition & Dietetics [Q1]
5	*International Journal of Pediatric Otorhinolaryngology*	19 (1.2)	1.3	Pediatrics/Otorhinolaryngology [Q3]
5	*Laryngoscope*	19 (1.2)	2.0	Otorhinolaryngology [Q2]
5	*Otolaryngology-Head and Neck Surgery*	19 (1.2)	2.5	Surgery/Otorhinolaryngology [Q1]
6	*Diabetes Obesity & Metabolism*	18 (1.1)	5.7	Endocrinology & Metabolism [Q1]
7	*American Journal of Ophthalmology*	17 (1.1)	4.2	Ophthalmology [Q1]
7	*Urology*	17 (1.1)	2.0	Urology & Nephrology [Q2]
8	*PLoS One*	16 (1.0)	2.6	Multidisciplinary Sciences [Q2]
9	*International Journal of Medical Sciences*	15 (1.0)	3.2	Medicine, General & Internal [Q1]
10	*Obesity Surgery*	14 (0.9)	3.1	Surgery [Q1]

JCR: Journal Citation Reports; JIF: Journal Impact Factor Q1, Q2, and Q3 indicate first, second, and third Journal Impact Factor quartiles, respectively. Journals with tied article counts were assigned the same rank. JCR category and quartile refer to the best-ranked category when a journal was assigned to more than one category.

## Data Availability

All bibliometric records analyzed in this study were retrieved from the Web of Science Core Collection and are available from the publisher under standard licensing.

## Supplementary Materials

The following supporting information can be downloaded at: https://www.mdpi.com/article/10.3390/healthcare14142143/s1, Figure S1: Annual publication output and mean citations per year in Scopus, 2018 to 2025. Figure S2: Clinical-domain and study-purpose distribution of TriNetX-based studies in Scopus. Table S1: Top publication sources of TriNetX-based articles in Web of Science and Scopus. File S1: R script for country, regional, and institutional productivity. File S2: VOSviewer thesaurus for author-keyword harmonization. File S3: R script for clinical-domain and study-purpose classification, domain × purpose gap mapping, and audit sampling.

## Abbreviations

The following abbreviations are used in this manuscript:

AKI	Acute kidney injury
CKD	Chronic kidney disease
COPD	Chronic obstructive pulmonary disease
COVID-19	Coronavirus disease 2019
DPP-4	Dipeptidyl peptidase-4
EHR	Electronic health record
GLP-1 RA	Glucagon-like peptide-1 receptor agonist
HIV	Human immunodeficiency virus
IBD	Inflammatory bowel disease
ICI	Immune checkpoint inhibitor
JCR	Journal Citation Reports
JIF	Journal Impact Factor
MASLD	Metabolic dysfunction-associated steatotic liver disease
MCP	Multiple-country publication
NHANES	National Health and Nutrition Examination Survey
NHIRD	National Health Insurance Research Database
Q	Journal quartile
RMSE	Root mean square error
RP	Reprint author (Web of Science field)
SARS-CoV-2	Severe acute respiratory syndrome coronavirus-2
SGLT2i	Sodium-glucose cotransporter 2 inhibitor
TS	Topic search (Web of Science search field)
WoS	Web of Science
