# Supplementary material for: Mapping TriNetX-Based Real-World Evidence Publications by Clinical Domain and Study Purpose, 2018–2025: A Bibliometric Analysis
Source: Healthcare (Basel). 2026 Jul 16;14(14):2143. doi: 10.3390/healthcare14142143 (PMC13409890; doi:10.3390/healthcare14142143)
Supplement: Supplementary file 1 [file healthcare-14-02143-s001.zip › Supplementary Table S1.pdf]

Table S1. Top publication sources of TriNetX-based articles in Web of Science and Scopus.

Web of Science			Scopus		
**Rank**	**Journal**	**Article count**	**Rank**		**Article count**
1	*Journal of Clinical Medicine*	32	1	*Journal of the American Academy of Dermatology*	45
2	*Journal of Medical Virology*	23	2	*Journal of Clinical Medicine*	32
3	*JAMA Network Open*	21	3	*Journal of Clinical Oncology*	28
3	*Scientific Reports*	21	4	*Journal of Medical Virology*	23
4	*Diabetes Research and Clinical Practice*	20	5	*Otolaryngology - Head and Neck Surgery (United States)*	22
5	*Frontiers in Nutrition*	19	6	*JAMA Network Open*	20
5	*International Journal of Pediatric Otorhinolaryngology*	19	6	*Diabetes Research and Clinical Practice*	20
5	*Laryngoscope*	19	7	*American Journal of Ophthalmology*	19
5	*Otolaryngology-Head and Neck Surgery*	19	7	*Scientific Reports*	19
6	*Diabetes Obesity & Metabolism*	18	7	*Frontiers in Nutrition*	19
7	*American Journal of Ophthalmology*	17	7	*Laryngoscope*	19
7	*Urology*	17	7	*International Journal of Pediatric Otorhinolaryngology*	19
8	*PLoS One*	16	8	*Diabetes, Obesity and Metabolism*	18
9	*International Journal of Medical Sciences*	15	9	*Urology*	17
10	*Obesity Surgery*	14	10	*Obesity Surgery*	15
-	-	-	10	*International Journal of Medical Sciences*	15
