# Supplementary figures and images for: Mapping TriNetX-Based Real-World Evidence Publications by Clinical Domain and Study Purpose, 2018–2025: A Bibliometric Analysis
Source: Healthcare (Basel). 2026 Jul 16;14(14):2143. doi: 10.3390/healthcare14142143 (PMC13409890; doi:10.3390/healthcare14142143)

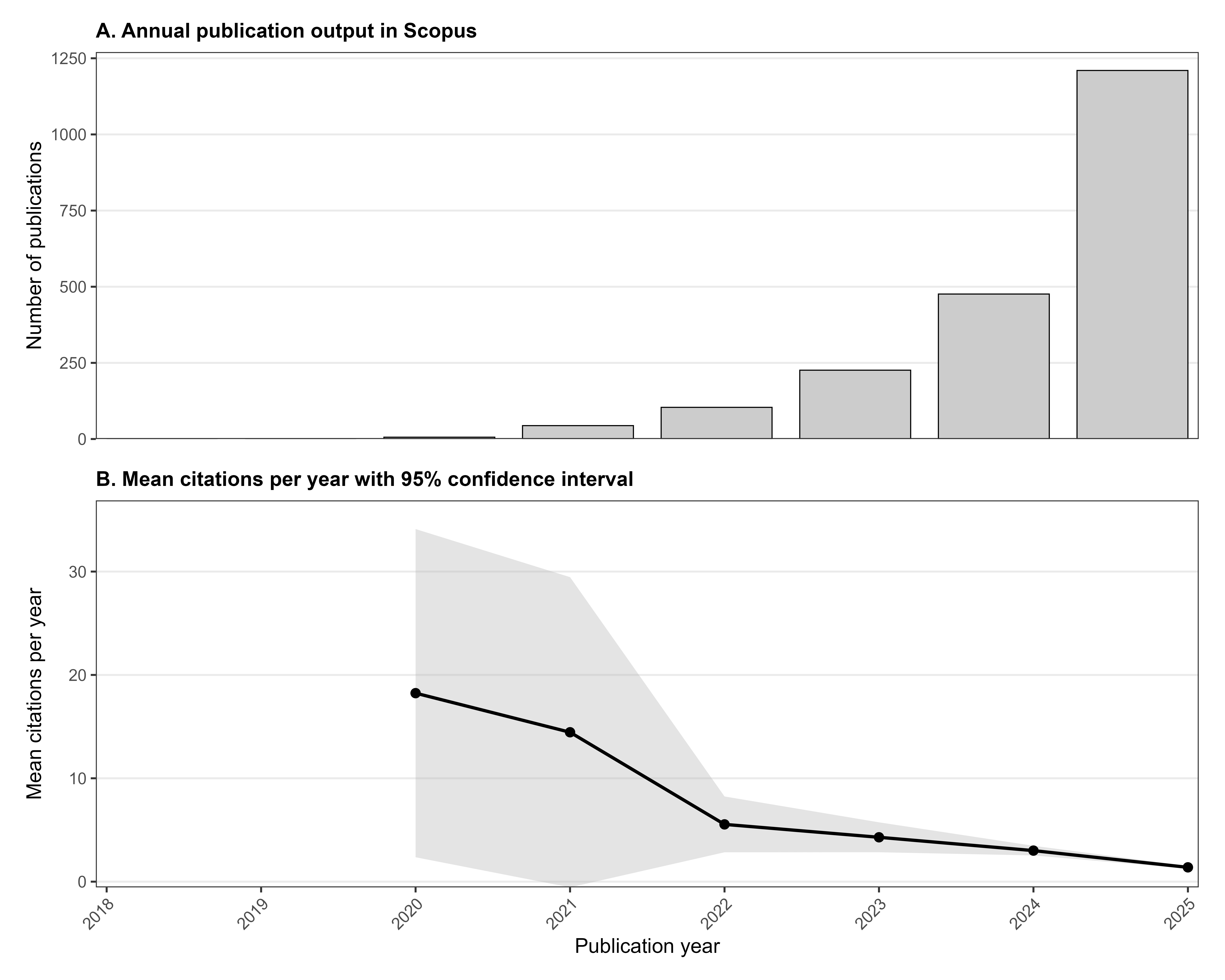

Supplement: Supplementary file 1 [file healthcare-14-02143-s001.zip › Supplementary Figure S1.png]

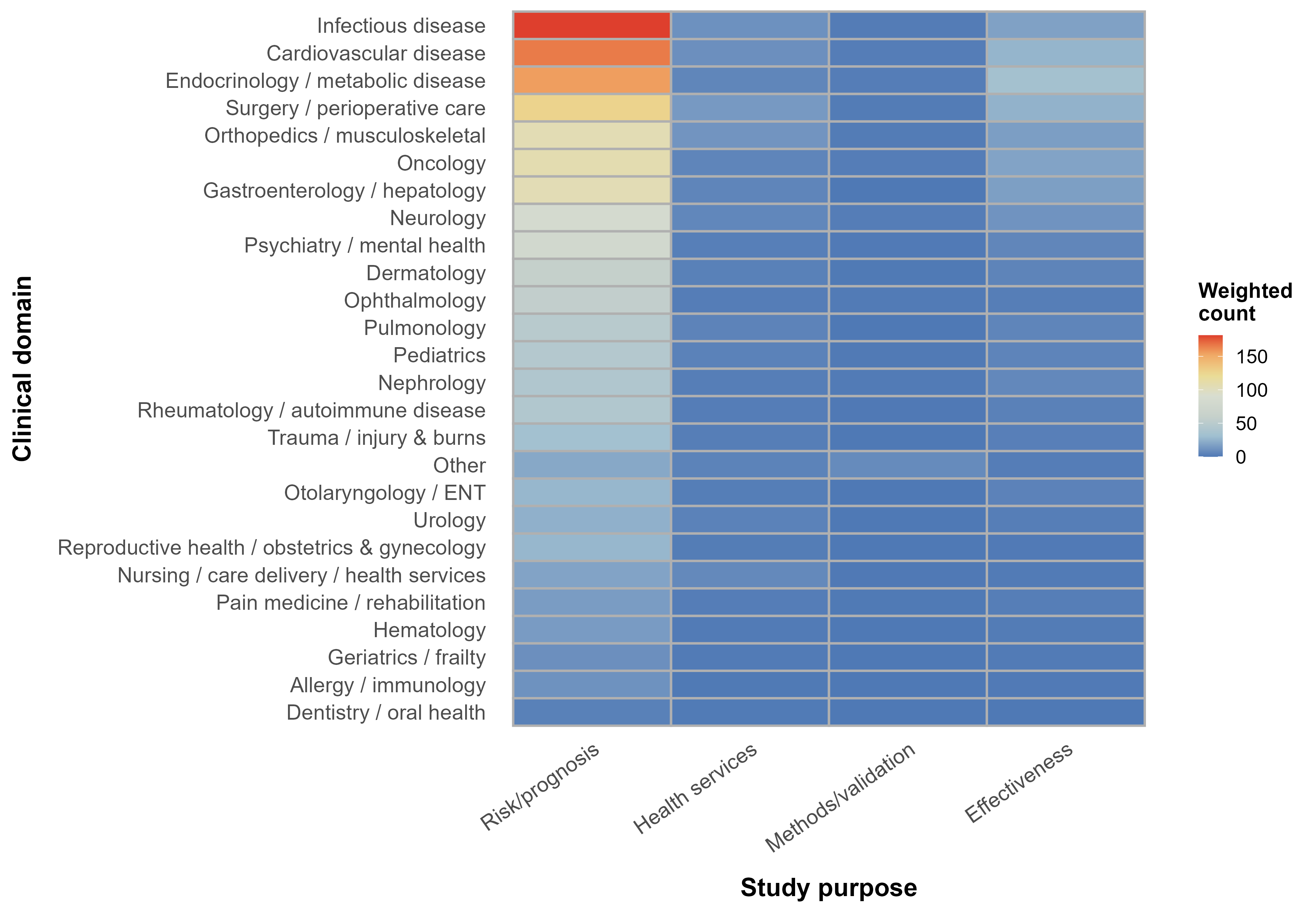

Supplement: Supplementary file 1 [file healthcare-14-02143-s001.zip › Supplementary Figure S2.png]
